# Anti-fatigue effects of dietary nucleotides in mice

**DOI:** 10.1080/16546628.2017.1334485

**Published:** 2017-06-14

**Authors:** Meihong Xu, Rui Liang, Yong Li, Junbo Wang

**Affiliations:** ^a^ Department of Nutrition and Food Hygiene, School of Public Health, Peking University, Beijing, PR China; ^b^ Beijing Key Laboratory of Toxicological Research and Risk Assessment for Food Safety, Peking University, Beijing, PR China​​; ^c^ Department of Nutrition, The First Affiliated hospital of Zhengzhou University, Zhengzhou, PR China

**Keywords:** Dietary nucleotides, anti-fatigue, forced swimming test

## Abstract

As the building blocks of nucleic acids, nucleotides are conditionally essential nutrients that exhibit multifaceted activities. The present study aimed to evaluate the anti-fatigue effects of dietary nucleotides (NTs) on mice and explore the possible underlying mechanism. Mice were randomly divided into four experimental sets to detect different indicators. Each set of mice was then divided into four groups: (i) one control group and (ii) three NTs groups, which were fed diets supplemented with NTs at concentrations of 0%, 0.04%, 0.16%, and 0.64% (wt/wt). NTs could significantly increase the forced swimming time, enhance lactate dehydrogenase activity and hepatic glycogen levels, as well as delay the accumulation of blood urea nitrogen and blood lactic acid in mice after 30 days of treatment. NTs also markedly improved fatigue-induced alterations in oxidative stress biomarkers and antioxidant enzymes. Notably, NTs increased the mitochondrial energy metabolic enzyme activities in the skeletal muscles of mice. These results suggest that NTs exert anti-fatigue effects, which may be attributed to the inhibition of oxidative stress and the improvement of mitochondrial function in skeletal muscles. NTs could be used as a novel natural agent for relieving exercise fatigue.

**Abbreviations**: ATP: adenosine triphosphate; BLA: blood lactic acid; GSH-Px: glutathione peroxidase; LDH: lactate dehydrogenase; MDA: malondialdehyde; NTs: dietary nucleotides; SDH: succinate dehydrogenase; SOD: superoxide dismutase; BUN: blood urea nitrogen

## Introduction

Fatigue is a feeling of extreme tiredness, which can result in a broad range of physical and mental unfitness, such as inattention, distraction, and drowsiness [1,2]. This condition mainly results from the depletion of energy sources, including the accumulation of end products of fatigue, disorder in the internal environment of the body, and decrease in glycemic levels and liver glycogen consumption [3]. Fatigue is a suboptimal health status and may be associated with various illnesses. With an accelerating pace of life and fierce social competition, fatigue has become a commonly occurring condition. Thus, efforts such as nutrition interventions are necessary to determine a safe and effective method for preventing fatigue.

Oxidative stress has been identified as one of the factors leading to fatigue [4]. High levels of oxidative stress lead to excessive generation of reactive oxygen species (ROS). These species are highly reactive molecules that cause lipid peroxidation in the membrane structure and damage the cellular structure. The release of ROS could result in lipid peroxidation in the mitochondrial membrane. Damaged mitochondria were found to reduce cellular respiration and adenosine triphosphate (ATP) generation; they are also among the primary causes of fatigue [5]. Interventions that reduce oxidative damage can effectively relieve fatigue, as suggested by study findings that antioxidants exert beneficial effects on fatigue [6,7]. Previous findings indicate that recovery from exercise-induced fatigue requires repair of the damage that has occurred in the body and/or prompts the elimination of metabolic products that have accumulated during exercise [8].

Dietary nucleotides (NTs) can be absorbed and used by all organs, which may benefit from an exogenous supply to save energy and optimize organ function. NTs have many beneficial functions, including antitumor acitivity, immune modulation, liver protective ability, and normalization of metabolism [9–12]. In addition, NTs exhibit superior antioxidant and anti-aging properties, as confirmed by our previous study [13]. However, studies on the anti-fatigue effects of NTs have rarely been reported. Thus, the present study was designed to evaluate the anti-fatigue activity of NTs and explore the possible underlying mechanism in mice.

## Materials and methods

### Materials and reagents

Basal diet (AIN-93G rodent diet) and the NTs-supplemented diet (basal diet supplemented with 0.4 g, 1.6 g and 6.4 g NTs*kg-1 respectively) were produced by HFK Bioscience Co. Ltd. (Beijing, China). NTs provided by Zhen-Ao Biotechnology Ltd. Co. (Dalian, China) were derived from brew yeast RNA. The NTs content was more than 99%. This product contained 22.8% 5ʹ-adenosine monophosphate (5ʹ-AMP), 26.6% 5ʹ-cytidine monophosphate (5ʹ-CMP), 20.4% 5ʹ-guanosine monophosphate (5ʹ-GMP) Na_2_, and 30.2% 5ʹ-uridine monophosphate (5ʹ-UMP) Na_2_. Dietary ingredients were thoroughly mixed in a mixture, made into pellets and air-dried at room temperature.

Assay kits used for the determination of blood urea nitrogen (BUN) and lactate dehydrogenase (LDH) were purchased from Yingkexinchuang Science and Technology Ltd. (Macau, China). The detection kits of blood lactic acid (BLA), hepatic glycogen, superoxide dismutase (SOD), glutathione peroxidase (GSH-Px), succinate dehydrogenase (SDH), Na^+^-K^+^-ATPase and Ca^2+^-Mg^2+^-ATPase activity, and malondialdehyde (MDA) were purchased from Nanjing Jiancheng Biotechnology Institute (Nanjing, China). All other reagents used in this study were of analytical grade.

### Animals and treatment

The present study, after approval from the Institutional Animal Care and Use Committee of Peking University (Ethical approval code: LA2015081, February 2015), used a total of 160 male ICR mice (6–8 weeks old, 18–22 g), which were procured from the Animal Service of Health Science Center, Peking University. They were housed at 25 ± 1◦C, 50–60% humidity, and maintained on a 12 h:12 h light–dark cycle, with free access to standard food and water. All animals were treated according to the Principles of Laboratory Animal Care (NIH publication No. 85–23, revised 1985) and the guidelines of Peking University Animal Research Committee.

After acclimatization for 1 week, the mice were randomly divided into four experimental sets (n = 40). Each set of mice were then divided into four groups (n = 10): control group, and three NTs intervention groups which were designated as a low-dose group (NTs-L), medium-dose group (NTs-M), and high-dose group (NTs-H). Control mice were fed with rodent diet (Vital River Ltd. Co., Beijing). Mice in the three experimental groups were fed with 0.01%, 0.16%, or 0.64% (wt/wt) NTs in the diet, respectively. The doses refer to the previous study in our laboratory [11–13]. Experimental mice were administrated by gavage for 30 days, and then were used for further experiments.

### Forced swimming test

Mice from Experimental Set 1 were used for the forced swimming test. Forced swimming test was carried out as described previously [3]. Briefly, 30 minutes after the final treatments, the mice were placed individually in a swimming pool filled with water (25 ± 1^◦^C) to a depth of 30 cm with a lead sheath (5% of the mouse’s body weight) attached to the tail root of each mouse. The swimming time was recorded immediately when the physical strength of the mouse was exhausted and it could not rise to the surface for more than 10 s.

### Biochemical assay

Mice from Experimental Set 2 were used for biochemical assay. Thirty minutes after the final oral administration, the mice were forced to swim in water at 30◦C for 90 min without any loads. After resting for an hour, a blood sample was obtained from the eyeballs and skeletal muscles (quadriceps femoris of both hind legs) of the mice. The serum was prepared by centrifugation at 2000 rpm at 4◦C for 15 min. The BUN content and LDH activity in serum were measured by automatic biochemical analyzer (Olympus Corporation, Tokyo, Japan). The SOD, GSH-Px, SDH, Na^+^K^+^-ATPase, and Ca^2+^-Mg^2+^-ATPase activity, and MDA levels in skeletal muscles were determined by detection kits according to the instructions.

### Determination of blood lactic acid

The concentrations of BLA were determined in mice from Experimental Set 3. Thirty minutes after the final oral administration, the mice were forced to swim in water at 30◦C for 10 min without any loads. Blood was obtained at three time points: at baseline, 0 min after swimming, and 20 min after swimming. A quantiaty of 20 µL blood was accurately collected from the angular vein of mice by glass capillary each time and then immediately moved into the bottom of a 5 mL centrifuge tube, which was joined with 0.48 ml 1% sodium fluoride solution in advance. The glass capillary was flushed with supernatant several times. The concentrations of BLA were determined according to the procedures provided by the kits. The area under the BLA curve (AUC) was calculated according to the following formula:

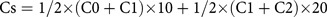

C0, C1, and C2 stand for the BLA concentration of mice at baseline, 0 and 20 min after swimming, respectively. Cs stands for the area under the BLA curve.

### Examination of hepatic glycogen

Mice from Experimental Set 4 were used to examine hepatic glycogen. Thirty minutes after the last administration of NTs, the mice were killed and their livers were immediately isolated and homogenized to 10% solution with normal saline at 4◦C. Hepatic glycogen levels were determined using available kits.

### Statistical analysis

The data were expressed as mean ± standard deviation (SD). Differences between groups were analyzed by one-way ANOVA test followed by Tukey’s post hoc least significant difference test if variances were equal or Tamhane’s T3 test if variances were not equal. p < 0.05 was considered significant.

## Results

### Effects of NTs on the body weight of mice

The effects of NTs on the body weight of mice during the experiment are shown in Table 1. The results showed that there was no statistical significance differences of body weight between the control and NTs groups in Experimental Set 1, 2, 3, and 4, respectively

**Table 1.** Effects of NTs on the body weight in mice.

Body weight (g)	Control		NTs-L		NTs-M		NTs-H	
	Mean	SD	Mean	SD	Mean	SD	Mean	SD
Set 1								
Initial body weight	19.40	0.76	19.08	1.06	19.11	1.10	19.46	1.20
Final weight	38.35	1.73	35.82	1.92	36.08	5.70	38.38	4.78
Set 2								
Initial body weight	18.72	1.06	18.31	1.63	18.53	1.20	19.22	1.09
Final weight	33.55	1.77	36.18	1.93	35.91	3.52	34.74	4.30
Set 3								
Initial body weight	20.01	1.20	19.02	2.00	19.99	1.75	20.50	2.42
Final weight	35.15	2.17	34.74	2.57	36.59	4.58	35.48	2.99
Set 4								
Initial body weight	19.32	1.44	18.72	1.81	18.94	1.58	19.40	1.90
Final weight	36.80	4.57	36.22	4.71	41.33	3.80	42.43	3.39

n* *= 10 for each group. NTs-L, dietary nucleotides low-dose group; NTs-M, dietary nucleotides medium-dose group; NTs-H, dietary nucleotides high-dose group

### Effects of NTs in the forced swimming test

**Figure 1.**
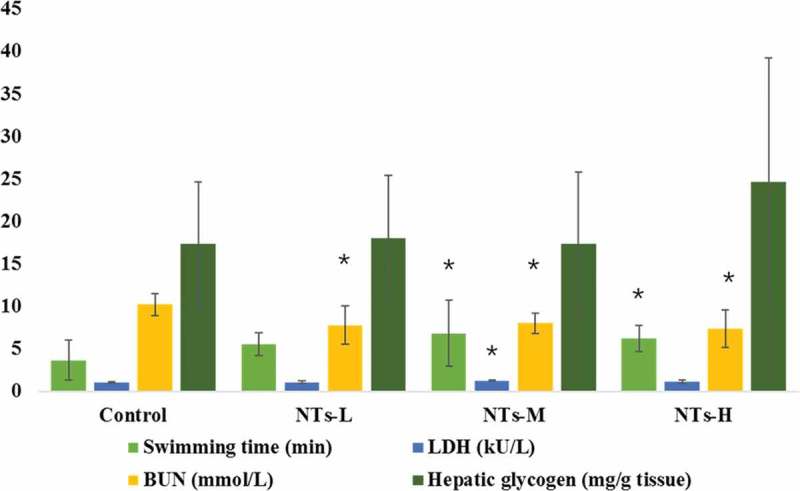
Effects of NTs on the forced swimming time, lactate dehydrogenase (LDH), serum urea nitrogen (SUN), and hepatic glycogen content in mice. Data were presented as means *±* SD (n = 10). * p < 0.05, versus control group. NTs-L, dietary nucleotides low-dose group; NTs-M, dietary nucleotides medium-dose group; NTs-H, dietary nucleotides high-dose group.

The effects of NTs on the forced swimming time of mice are shown in Figure 1. As expected, in comparison with the control group, the forced swimming time in all three NTs groups was longer and the difference was statistically significant in the NTs-M and NTs-H (p < 0.05). In general, when compared to the control group, the forced swimming time in the NTs-L, NTs-M, and NTs-H increased by 51.23%, 86.57%, and 71.23%, respectively.

### Effects of NTs on lactate dehydrogenase (LDH), blood urea nitrogen (BUN) and hepatic glycogen content in mice

As shown in Figure 1, compared with the control group, the LDH activity was significantly increased in NTs-M (p < 0.05) and the BUN levels were markedly decreased in all three NTs-treated groups (p < 0.05). However, the hepatic glycogen levels of mice were improved in NTs groups without remarkable differences in the comparison with control group (p > 0.05), which indicates that NTs had no effect on glycogen levels.

### Effects of NTs on blood lactic acid (BLA) levels in mice

**Figure 2.**
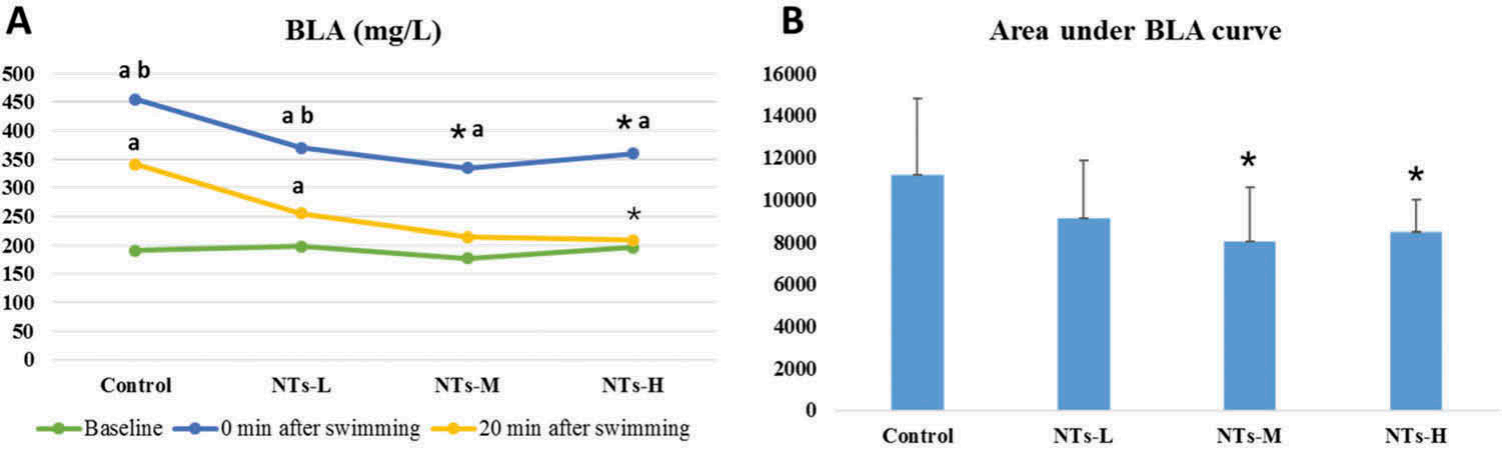
Effects of NTs on the content of BLA at different time points and the area under BLA curve in mice. Data were presented as means *±* SD (n = 10). * p < 0.05, versus control group; ^a^ p < 0.05, versus baseline; ^b^ p < 0.05, versus 20 min after swimming. NTs-L, dietary nucleotides low-dose group; NTs-M, dietary nucleotides medium-dose group; NTs-H, dietary nucleotides high-dose group.

The results about the effects of NTs on BLA in mice at different time points are shown in Figure 2. There were no significant differences among the groups at baseline. The BLA levels of 0 min after swimming were remarkable increased in comparison with baseline in all the groups (p < 0.05). Similarly, compared with the baseline, there was significantly differences between baseline and 20 min after swimming, in the control and NTs-L group (p < 0.05). Compared with the control group, the concentrations of BLA in NTs-M and NTs-H were significantly decreased at 0 min after swimming (p < 0.05). At 20 min after swimming, the concentrations of BLA in NTs-H group was significantly decreased (p < 0.05). After NTs treatment, the area under the BLA curve (AUC) was also reduced in comparison with the control group (p < 0.05 for NTs-M and NTs-H).

### Effects of NTs on parameters of oxidative stress in skeletal muscles of mice

The SOD, GSH-Px activity, and MDA levels are shown in Table 2 to evaluate the level of oxidative stress in skeletal muscles of mice. After the treatment, the activities of SOD and GSH-Px, in NTs-M and NTs-H group, were significantly improved in the comparison with control group (p < 0.05). In addition, MDA levels in skeletal muscle were significantly attenuated in NTs groups (p < 0.05) compared with the control group.

**Table 2.** Effects of NTs on SOD, GSH-Px activity, and MDA levels in skeletal muscles of mice.

Parameters	Control		NTs-L		NTs-M		NTs-H	
	Mean	SD	Mean	SD	Mean	SD	Mean	SD
SOD (U/mg pro)	96.10	23.73	103.10	17.58	120.83*	10.00	108.77	21.50
GSH-Px (U/g pro)	1.19	0.12	1.27	0.18	1.43*	0.09	1.35*	0.05
MDA (nmol/mg pro)	3.72	1.47	1.66*	1.17	2.80*	1.15	2.24*	1.01

n* *= 10 for each group. NTs-L, dietary nucleotides low-dose group; NTs-M, dietary nucleotides medium-dose group; NTs-H, dietary nucleotides high-dose group

### Effect of NTs on activities of mitochondrial energy metabolic enzyme in skeletal muscles of mice

The SDH, Na^+^-K^+^-ATPase, and Ca^2+^-Mg^2+^-ATPase activity are shown in Table 3 to evaluate the level of Mitochondrial Energy Metabolic enzyme in skeletal muscles of mice. After the treatment, the activity of SDH and Ca^2+^-Mg^2+^-ATPase was significantly improved in NTs-M (p < 0.05). Similarly, the activity of Na^+^-K^+^-ATPase in skeletal muscle were significantly increased in NTs-M and NTs-H groups (p < 0.05) compared with the control group.

**Table 3.** Effects of NTs on SDH, Na+-K+-ATPase and Ca2+ -Mg2+-ATPase activity in skeletal muscles of mice.

Parameters	Control		NTs-L		NTs-M		NTs-H	
	Mean	SD	Mean	SD	Mean	SD	Mean	SD
SDH (U/mg pro)	0.81	0.08	0.90	0.06	0.96*	0.06	0.84	0.05
Na+K+-ATPase (U/mg pro)	1.02	0.12	1.16	0.09	1.27*	0.15	1.14*	0.13
Ca2+ -Mg2+-ATPase (U/mg pro)	0.53	0.10	0.67	0.09	0.70*	0.11	0.66	0.03

*n *= 10 for each group. NTs-L, dietary nucleotides low-dose group; NTs-M, dietary nucleotides medium-dose group; NTs-H, dietary nucleotides high-dose group

## Discussion

With their multifaceted activities, NTs have gained increasing popularity as dietary supplements. A number of reports have demonstrated that adding NTs to dietary formulas increases the production of immunoglobulins, improves response to vaccines, reduces morbidity, and increases tolerance to dietary antigens [12,14]. Our previous studies found that NTs are not toxic or carcinogenic in rats at a concentration of up to 0.64% (of body weight) for their entire lifetime, and could extend the lifespan in SD rats in a dose-dependent manner [13]. To the best of our knowledge, the present study is the first to report that dietary NTs supplementation improves the fatigue. We also found that NTs could increase forced swimming time, LDH activity, and hepatic glycogen levels, simultaneously, NTs could decrease the contents of BUN and BLA in mice. The anti-fatigue effect may be associated with the inhibition of oxidative stress and improvement of mitochondrial activity.

Repetitive and sustained physical labor results in fatigue, provoking systemic alterations, including endocrine, immune, and metabolic dysfunction [15]. The use of forced swimming tests provides a satisfactory experimental model for evaluating anti-fatigue activities in mice [16]. In the present study, NT treatment prolonged the time to exhaustion of the mice, particularly at 0.16% and 0.64% NT-treated groups, indicating the anti-fatigue effects of NTs on mice. To further study the anti-fatigue property of NTs, several biochemical markers for fatigue were measured, including BUN, LDH, BLA, and hepatic glycogen. BUN is formed in the liver as a metabolic product of protein and amino acid; it is one of the blood biochemical indexes related to fatigue. With increased exercise, the energy from sugar and fat catabolism becomes insufficient for the body; proteins and amino acids exhibit a stronger catabolism to compensate for the energy consumption, which causes an increase in BUN [17]. A remarkable positive correlation is observed between the level of BUN and the degree of fatigue [18]. During prolonged exercises, excess lactic acid is generated and accumulated in skeletal muscles, leading to muscle fatigue [19]. Therefore, BLA can be used as a fatigue index. In addition, glycogen is an important energy material that enables movement and provides adequate energy for muscle contraction. Energy use reduces glycogen; meanwhile, an increase in hepatic glycogen can improve exercise endurance [20]. In the present study, NTs can increase the LDH activity and the hepatic glycogen levels, as well as decrease the contents of BUN and BLA in mice.

High energy consumption during intense exercise may cause an imbalance between the oxidation and anti-oxidation systems, resulting in an increase in ROS and a reduction in antioxidant activities. These behaviors lead to enhanced ROS production. Oxidative stress is involved in both chronic fatigue and other fatigue-related disorders [21]. Extreme physical stress could lead to excessive generation of ROS in the skeletal muscle which, in turn, results in peripheral fatigue [22,23]. SOD, GSH-Px activity, and MDA levels, which generally indicate the capacity of the antioxidant defense system, were measured to evaluate the antioxidant activity of NTs. SOD and GSH-Px are important enzymatic antioxidant systems for scavenging free radicals and their metabolites [24]. MDA is one of the degradation products of lipid peroxidation, an important indicator for evaluating cellular oxidative stress [25]. Studies indicated that NTs exhibit remarkable anti-oxidative activities [11,13]. Our results suggested that the anti-fatigue effects of NTs are closely related to the protection of corpuscular membrane by improving the activities of several enzymes and preventing lipid oxidation.

In the present study, mitochondrial function was improved in the skeletal muscles of mice after NT treatment. Continuous ATP generation is required in myocytes to maintain prolonged physical activity. The mitochondrion is an important intracellular organelle in eukaryotic cells, which is the main venue of oxidative phosphorylation and ATP production in mammalian cells. Moreover, the mitochondrion plays an important mediating role for oxidative stress [26]. Consequently, mitochondrial function in skeletal muscles contributes to exercise-induced fatigue. In the present study, the activities of SDH, Na^+^-K^+^-ATPase, and Ca^2+^-Mg2^+^-ATPase were measured to evaluate mitochondrial function. Energy metabolism includes anabolism and catabolism, involving many biological enzymes [27]. Na^+^-K^+^-ATPase and Ca^2+^-Mg^2+^-ATPase are the two main ATP degradation enzymes, which can hydrolyze ATP to supply direct free energy [28]. It plays an important role in maintaining the physiologic functions of material transport, energy conversion, and information transmission [29]. Na^+^-K^+^-ATPase and Ca^2+^-Mg^2+^-ATPase are among the main factors responsible for fatigue [30–32]. In addition, SDH is a rate-limiting enzyme associated with the regulation of the glycolytic pathway and the Krebs cycle and the catalysis of ATP synthesis [27]. The activities of these enzymes may be important in energy metabolism in the skeletal muscle under fatigue. Under normal conditions, enzymatic activities are regulated to maintain the balance between anabolism and catabolism. Under fatigue conditions, low levels of SDH, Na^+^-K^+^-ATPase, and Ca^2+^Mg^2+^-ATPase activity in the skeletal muscle were observed. This finding indicated that ATP hydrolysis occurred, signifying mitochondrial damage, and that balance was lost, owing to the decreased levels of Na^+^-K^+^-ATPase and Ca^2+^-Mg^2+^-ATPase activity. However, in the present study, we found that NTs could improve mitochondrial function in the skeletal muscles of mice by enhancing the activities of energy metabolic enzymes, such as SDH, Na^+^-K^+^-ATPase, and Ca^2+^-Mg^2+^-ATPase, thereby suppressing oxidative stress and generating more ATP for energy supplementation [33,34].

## Conclusions

Our combined results demonstrated for the first time that NTs exert anti-fatigue effects. NTs could increase the forced swimming time of mice by enhancing LDH activity and hepatic glycogen levels and by delaying the accumulation of BUN and BLA. NTs could also improve mitochondrial function and inhibit oxidative stress in the skeletal muscles of mice, which may be an action pathway of its anti-fatigue effects. NTs could be used as a novel natural agent for alleviating exercise fatigue. Further research *in vitro* is required to explore the exact molecular mechanism by which NTs play their role in anti-fatigue effects.

## References

[1] Moriura T, Matsuda H, Kubo M. Pharmacological study on Agkistrodon blomhoffii blomhoffii BOIE. V. anti-fatigue effect of the 50% ethanol extract in acute weight-loaded forced swimming-treated rats. Biol Pharm Bull. 1996;19(1):62–7.​​ 8820913 10.1248/bpb.19.62

[2] Kim KM, Yu KW, Kang DH, et al Anti-stress and anti-fatigue effects of fermented rice bran. Biosci Biotechnol Biochem. 2001;65(10):2294–2296. 11758925 10.1271/bbb.65.2294

[3] Tan W1, Yu KQ, Liu YY, et al Anti-fatigue activity of polysaccharides extract from Radix Rehmanniae Preparata. Int J Biol Macromol. 2012;50(1):59–62. 21983027 10.1016/j.ijbiomac.2011.09.019

[4] Azizbeigi K, Stannard SR, Atashak S, et al Antioxidant enzymes and oxidative stress adaptation to exercise training: comparison of endurance, resistance, and concurrent training in untrained males. J Exerc Sci Fitness. 2014;12(1):1–6.

[5] Echtay KS, Roussel D, St-Pierre J, et al Superoxide activates mitochondrial uncoupling proteins. Nature. 2002;415(6867):96–99. 11780125 10.1038/415096a

[6] Wang X, Xing R, Chen Z, et al Effect and mechanism of mackerel (Pneumatophorus japonicus) peptides for anti-fatigue. Food Funct. 2014;5(9):2113–2119. 25002163 10.1039/c4fo00121d

[7] Lee J-S, Kim H-G, Han J-M, et al Anti-fatigue effect of Myelophil in a chronic forced exercise mouse model. Eur J Pharmacol. 2015;764:100–108. 26142828 10.1016/j.ejphar.2015.06.055

[8] Chi A, Li H, Kang C, et al Anti-fatigue activity of a novel polysaccharide conjugates from Ziyang green tea. Int J Biol Macromol. 2015;80:566–572. 26141387 10.1016/j.ijbiomac.2015.06.055

[9] Martinez-Puig D, Manzanilla EG, Morales J, et al Dietary nucleotide supplementation reduces occurrence of diarrhoea in early weaned pigs. Livest Sci. 2007;108:276–279.

[10] Cai X, Bao L, Wang N, et al Dietary nucleotides supplementation and liver injury in alcohol-treated rats: a metabolomics investigation. Molecules. 2016;21(4):435. 27043516 10.3390/molecules21040435 PMC6273469

[11] Cai X, Bao L, Wang N, et al Dietary nucleotides protect against alcoholic liver injury by attenuating inflammation and regulating gut microbiota in rats. Food Funct. 2016;7(6):2898–2908. 27247978 10.1039/c5fo01580d

[12] Xu M, Zhao M, Yang R, et al Effect of dietary nucleotides on immune function in Balb/C mice. Int Immunopharmacol. 2013;17(1):50–56. 23669334 10.1016/j.intimp.2013.04.032

[13] Xu M, Liang R, Guo Q, et al Dietary nucleotides extend the life span in Sprague-Dawley rats. J Nutr Health Aging. 2013;17(3):223–229. 23459974 10.1007/s12603-012-0399-z PMC12878984

[14] Che L, Hu L, Liu Y, et al Dietary nucleotides supplementation improves the intestinal development and immune function of neonates with intra-uterine growth restriction in a pig model. PLoS One. 2016;11(6):e0157314. 27304828 10.1371/journal.pone.0157314 PMC4909294

[15] Chaudhuri A, Behan PO Fatigue in neurological disorders. Lancet. 2004;363(9413):978–988. 15043967 10.1016/S0140-6736(04)15794-2

[16] You L, Ren J, Yang B, et al Antifatigue activities of loach protein hydrolysates with different antioxidant activities. J Agric Food Chem. 2012;60(50):12324–12331. 23136870 10.1021/jf3037825

[17] Li X, Zhang H, Xu H Analysis of chemical components of shiitake polysaccharides and its anti-fatigue effect under vibration. Int J Biol Macromol. 2009;45(4):377–380. 19643126 10.1016/j.ijbiomac.2009.07.005

[18] Huang W-C, Chiu W-C, Chuang H-L, et al. Effect of curcumin supplementation on physiological fatigue and physical performance in mice. Nutrients. 2015;7(2):905–921. 25647661 10.3390/nu7020905 PMC4344567

[19] Gibson H, Edwards RH Muscular exercise and fatigue. Sports Med. 1985;2(2):120–132. 3847097 10.2165/00007256-198502020-00004

[20] Anand T, Phani Kumar G, Pandareesh MD, et al Effect of bacoside extract from Bacopa monniera on physical fatigue induced by forced swimming. Phytother Res. 2012;26(4):587–593. 21959990 10.1002/ptr.3611

[21] Barclay JK, Hansel M Free radicals may contribute to oxidative skeletal muscle fatigue. Can J Physiol Pharmacol. 1991;69(2):279–284. 2054745 10.1139/y91-043

[22] Allen DG, Lamb GD, Westerblad H Skeletal muscle fatigue: cellular mechanisms. Physiol Rev. 2008;88(1):287–332. 18195089 10.1152/physrev.00015.2007

[23] Westerblad H, Allen DG, Lännergren J Muscle fatigue: lactic acid or inorganic phosphate the major cause? News Physiol Sci. 2002;17:17–21. 11821531 10.1152/physiologyonline.2002.17.1.17

[24] Elias RJ, Kellerby SS, Decker EA Antioxidant activity of proteins and peptides. Crit Rev Food Sci Nutr. 2008;48(5):430–441. 18464032 10.1080/10408390701425615

[25] Bagis S, Tamer L, Sahin G, et al Free radicals and antioxidants in primary ﬁbromyalgia: an oxidative stress disorder? Rheumatol Int. 2005;25(3):188–190. 14689230 10.1007/s00296-003-0427-8

[26] Sivitz WI, Yorek MA Mitochondrial dysfunction in diabetes: from molecular mechanisms to functional signiﬁcance and therapeutic opportunities. Antioxid Redox Signal. 2010;12(4):537–577. 19650713 10.1089/ars.2009.2531 PMC2824521

[27] Kolling J, Scherer EB, Siebert C, et al Homocysteine induces energy imbalance in rat skeletal muscle: is creatine a protector? Cell Biochem Funct. 2013;31(7):575–584. 23225327 10.1002/cbf.2938

[28] Huang X-P, Tan H, Chen B-Y, et al. Astragalus extract alleviates nerve injury after cerebral ischemia by improving energy metabolism and inhibiting apoptosis. Biol Pharm Bull. 2012;35(4):449–454. 22466546 10.1248/bpb.35.449

[29] Scheiner-Bobis G The sodium pump. Its molecular properties and mechanics of ion transport. Eur J Biochem. 2002;269(10):2424–2433. 12027879 10.1046/j.1432-1033.2002.02909.x

[30] Leppik JA, Aughey RJ, Medved I, et al Prolonged exercise to fatigue in humans impairs skeletal muscle Na+-K+-ATPase activity, sarcoplasmic reticulum Ca2+ release, and Ca2+ uptake. J Appl Physiol (1985). 2004;97(4):1414–1423. 15155714 10.1152/japplphysiol.00964.2003

[31] Chauhan VP, Tsiouris JA, Chauhan A, et al Increased oxidative stress and decreased activities of Ca(2+)/Mg(2+)-ATPase and Na(+)/K(+)-ATPase in the red blood cells of the hibernating black bear. Life Sci. 2002;71(2):153–161. 12031685 10.1016/s0024-3205(02)01619-3

[32] Fraser SF, Li JL, Carey MF, et al Fatigue depresses maximal in vitro skeletal muscle Na(+)-K(+)-ATPase activity in untrained and trained individuals. J Appl Physiol (1985). 2002;93(5):1650–1659. 12381750 10.1152/japplphysiol.01247.2001

[33] Juel C Oxidative stress (glutathionylation) and Na,KATPase activity in rat skeletal muscle. PLoS One. 2014;9(10):e110514. 25310715 10.1371/journal.pone.0110514 PMC4195747

[34] Srikanthan K, Shapiro JI, Sodhi K, The role of Na/K-ATPase signaling in oxidative stress related to obesity and cardiovascular disease. Molecules. 2016;21(9):1172 pii: E1172. 10.3390/molecules21091172 PMC5642908 27598118

